# Biomarker-driven stratification of disease-risk in non-metastatic medulloblastoma: Results from the multi-center HIT-SIOP-PNET4 clinical trial

**DOI:** 10.18632/oncotarget.5149

**Published:** 2015-09-05

**Authors:** Steven C. Clifford, Birgitta Lannering, Ed C. Schwalbe, Debbie Hicks, Kieran O' Toole, Sarah Leigh Nicholson, Tobias Goschzik, Anja zur Mühlen, Dominique Figarella-Branger, François Doz, Stefan Rutkowski, Göran Gustafsson, Torsten Pietsch

**Affiliations:** ^1^ Northern Institute for Cancer Research, Newcastle University, Newcastle upon Tyne, United Kingdom; ^2^ Department of Pediatrics, University of Gothenburg and The Queen Silvia Children's Hospital, Gothenburg, Sweden; ^3^ Department of Applied Sciences, Northumbria University, Newcastle upon Tyne, United Kingdom; ^4^ Department of Neuropathology, University of Bonn, Bonn, Germany; ^5^ Department of Pathology and Neuropathology, Assistance Publique Hôpitaux de Marseille, Aix Marseille University, Marseille, France; ^6^ Institut Curie and University Paris Descartes, Paris, France; ^7^ University Medical Center Hamburg-Eppendorf, Hamburg, Germany; ^8^ Karolinska Institute, Stockholm, Sweden

**Keywords:** medulloblastoma, clinical trial, biomarker, stratification

## Abstract

**Purpose:**

To improve stratification of risk-adapted treatment for non-metastatic (M0), standard-risk medulloblastoma patients by prospective evaluation of biomarkers of reported biological or prognostic significance, alongside clinico-pathological variables, within the multi-center HIT-SIOP-PNET4 trial.

**Methods:**

Formalin-fixed paraffin-embedded tumor tissues were collected from 338 M0 patients (>4.0 years at diagnosis) for pathology review and assessment of the WNT subgroup (MB_WNT_) and genomic copy-number defects (chromosome 17, *MYC/MYCN*, 9q22 (*PTCH1*) and DNA ploidy). Clinical characteristics were reviewed centrally.

**Results:**

The favorable prognosis of MB_WNT_ was confirmed, however better outcomes were observed for non-MB_WNT_ tumors in this clinical risk-defined cohort compared to previous disease-wide clinical trials. Chromosome 17p/q defects were heterogeneous when assessed at the cellular copy-number level, and predicted poor prognosis when they occurred against a diploid (ch17(im)/diploid(cen)), but not polyploid, genetic background. These factors, together with post-surgical tumor residuum (R+) and radiotherapy delay, were supported as independent prognostic markers in multivariate testing. Notably, *MYC* and *MYCN* amplification were not associated with adverse outcome. In cross-validated survival models derived for the clinical standard-risk (M0/R0) disease group, (ch17(im)/diploid(cen); 14% of patients) predicted high disease-risk, while the outcomes of patients without (ch17(im)/diploid(cen)) did not differ significantly from MB_WNT_, allowing re-classification of 86% as favorable-risk.

**Conclusion:**

Biomarkers, established previously in disease-wide studies, behave differently in clinically-defined standard-risk disease. Distinct biomarkers are required to assess disease-risk in this group, and define improved risk-stratification models. Routine testing for specific patterns of chromosome 17 imbalance at the cellular level, and MB_WNT_, provides a strong basis for incorporation into future trials.

## INTRODUCTION

Medulloblastoma is the most common malignant brain tumor in children. Risk-adapted therapeutic protocols in non-infant patients encompass maximal surgical resection, cranio-spinal radiotherapy and chemotherapy. Treatment groups and intensity are defined by the presence (‘high-risk’ disease) or absence (‘standard-risk’) of clinical features associated with a poor prognosis; metastatic disease at diagnosis and/or significant post-operative tumor residues, and this stratification currently forms the basis of patient selection into clinical trials [1].

Recent advances in the biological sub-classification of medulloblastoma are leading to the conception of clinical trials aimed at more precise therapeutic stratification and improved outcomes [1]. Historical studies have identified biomarkers consistently associated with favorable (β-catenin nuclear immunopositivity as a marker of the WNT medulloblastoma molecular subgroup (MB_WNT_)) and poor (large-cell/anaplastic (LCA) pathology, *MYC* gene family amplification) prognosis [2–9], and their retrospective evaluation in the SIOP-UKCCSG-PNET3 clinical trial has validated their use alongside clinical factors for the improved definition of disease risk-stratification groups in disease-wide studies of non-infant medulloblastoma [10]. These stratification schemes will now form the basis of treatment selection in contemporary international clinical trials [1]. Additionally, further biomarkers with potential prognostic value, most notably the discovery of the four consensus medulloblastoma molecular subgroups (MB_WNT_, Sonic hedgehog (MB_SHH_), Group 3 (MB_Group3_) and Group 4 (MB_Group4_)), are emerging from recent research studies on retrospective cohorts of medulloblastoma patients [1, 11–14].

The validation of novel biomarkers and risk-stratification schemes in clinically-controlled cohorts is thus essential to their clinical application. Moreover, the specific therapeutic regimens used may potentially impact the prognostic significance of specific biomarkers, and validation of their relevance within the defined treatment groups used in current clinical trials (i.e. the clinical standard- or high-risk disease groups) is necessary. This will require large-scale and coordinated international studies.

Here, we report the first European prospective study of medulloblastoma biomarkers, undertaken as part of the multi-center HIT-SIOP-PNET4 trial (2001–2006), which enrolled 338 children from 120 centres, with clinically-defined non-metastatic, standard-risk medulloblastoma [15]. Sufficient FFPE tumor material was collected for prospective assay (2004–2010) of a selected panel of biomarkers of previously reported biological or prognostic significance (i.e. in ≥2 published series). The study aimed to (i) improve the early identification of the ∼20% patients with standard-risk medulloblastoma which cannot be cured by current treatment concepts, and (ii) identify patients with a favorable prognosis who may qualify for a controlled reduction of adjuvant treatment schemes.

## RESULTS

### HIT-SIOP-PNET4: Clinical and treatment-related factors

338 patients, aged 4 to 21 at diagnosis, were enrolled and their clinical characteristics have been reported previously [15]. In summary, male patients predominated (211 male, 127 female) and the five-year EFS (all patients, including R+ disease) was 79 ± 2%. Features significantly associated with reduced EFS in univariate analysis in the clinical study were: (i) R+ disease (31/317 (9.8%); *p* = 0.020), and (ii) a delay to the start of radiotherapy (≥49 days after surgery (30/335 (9.0%); *p* = 0.050) or as a continuous variable (*p* = 0.025)). Patient gender and age at diagnosis were not associated with EFS [15]. Histopathological review was completed for 336/338 patients, and identified 273 CMB (81%), 47 DMB (14%) and 16 LCA (5%) tumors. There was no survival difference between CMB and DMB patients. The 16 patients with LCA subtype tumors enrolled on the study prior to amendment showed a higher frequency of relapses, but these did not reach significance [15]. The exclusion of further LCA patients, together with M+ patients, from the HIT-SIOP-PNET4 cohort, as well as sharpening of the definitions of DMB and LCA in the revised WHO classification of tumors of the CNS in 2007 [16] may account for any variation in the distribution of histopathological variants compared to previously-reported non-infant disease-wide trials (i.e. 71 CMB (61%), 22 DMB (19%) and 23 LCA (20%) in SJMB96 [3]; 174 CMB (84%), 14 DMB (7%) and 19 LCA (9%) in SIOP-UKCCSG-PNET3 [10]). Data are summarized in Table 1.

**Table 1:** Clinical, pathological and molecular characteristics of the HIT-SIOP-PNET4 cohort (*n* = 338; all patients with available data are shown), and univariate (Cox proportional hazards) analysis of their prognostic associations

Variable	Categories	*n*	Five-year pEFS ±SE	Univariate Hazard Ratio (± CI)	*p*-value
^+^**Gender**	Male (M) Female (F) Ratio (M:F)	211 127 1.66:1	0.79 ± 0.03 0.80 ± 0.04	1.0 0.85 (0.52–1.41)	0.533
Age*	Median (years) Min.-Max.	9.0 3–20	–	0.99 (0.93–1.05)	0.754
^+^Pathology**	All others LCA	320 16	0.80 ± 0.02 0.64 ± 0.14	1.0 1.76 (0.71–4.37)	0.262
^+^**Residual tumor**	≤1.5 cm^2^ >1.5 cm^2^	286 31	0.82 ± 0.02 0.64 ± 0.09	1.0 2.34 (1.22–4.50)	**0.020**
^+^**Time from diagnosis to radiotherapy**	<49 days ≥49 days	305 30	0.81 ± 0.02 0.67 ± 0.09	1.0 1.93 (0.99–3.79)	**0.050**
Time from diagnosis to radiotherapy*	Median (days) Min.-Max.	35 15–92	–	1.03 (1.00–1.05)	**0.025**
**β-catenin nuclear accumulation**	No Yes	196 58	0.75 ± 0.03 0.91 ± 0.04	1.0 0.40 (0.17–0.94)	**0.019**
***CTNNB1* mutation**	No Yes	164 31	0.75 ± 0.04 0.89 ± 0.06	1.0 0.37 (0.12–1.21)	0.058
***MYC*/*MYCN* amplification (PCR)**	No Yes	160 23	0.79 ± 0.03 0.72 ± 0.09	1.0 1.26 (0.53–3.00)	0.606
***MYC* amplification (iFISH)**	No Yes	157 4	0.81 ± 0.03 1.00	1.0 0.22 (0.02–29.16)	0.542
***MYCN* amplification (iFISH)**	No Yes	147 13	0.82 ± 0.03 0.77 ± 0.12	1.0 1.41 (0.42–4.67)	0.588
**17p loss and/or 17q gain (diploid(cen)) (iFISH)**	No Yes	127 24	0.85 ± 0.03 0.57 ± 0.10	1.0 3.12 (1.44–6.76)	**0.007**
**Polyploid**	No Yes	72 85	0.78 ± 0.05 0.83 ± 0.04	1.0 0.81 (0.39–1.68)	0.572
***PTCH1* (9q22) loss**	No Yes	138 13	0.80 ± 0.03 0.85 ± 0.10	1.0 0.82 (0.19–3.46)	0.781

* Assessed as a continuous variable.

** Patients with LCA tumors recruited prior to study amendment in November 2003. pEFS, event-free survival probability; SE, standard error; CI, 95% confidence interval. Significant prognostic associations are marked in bold type.

+ Previously reported data shown for information [15].

### MB_WNT_ subgroup

**Figure 1:**
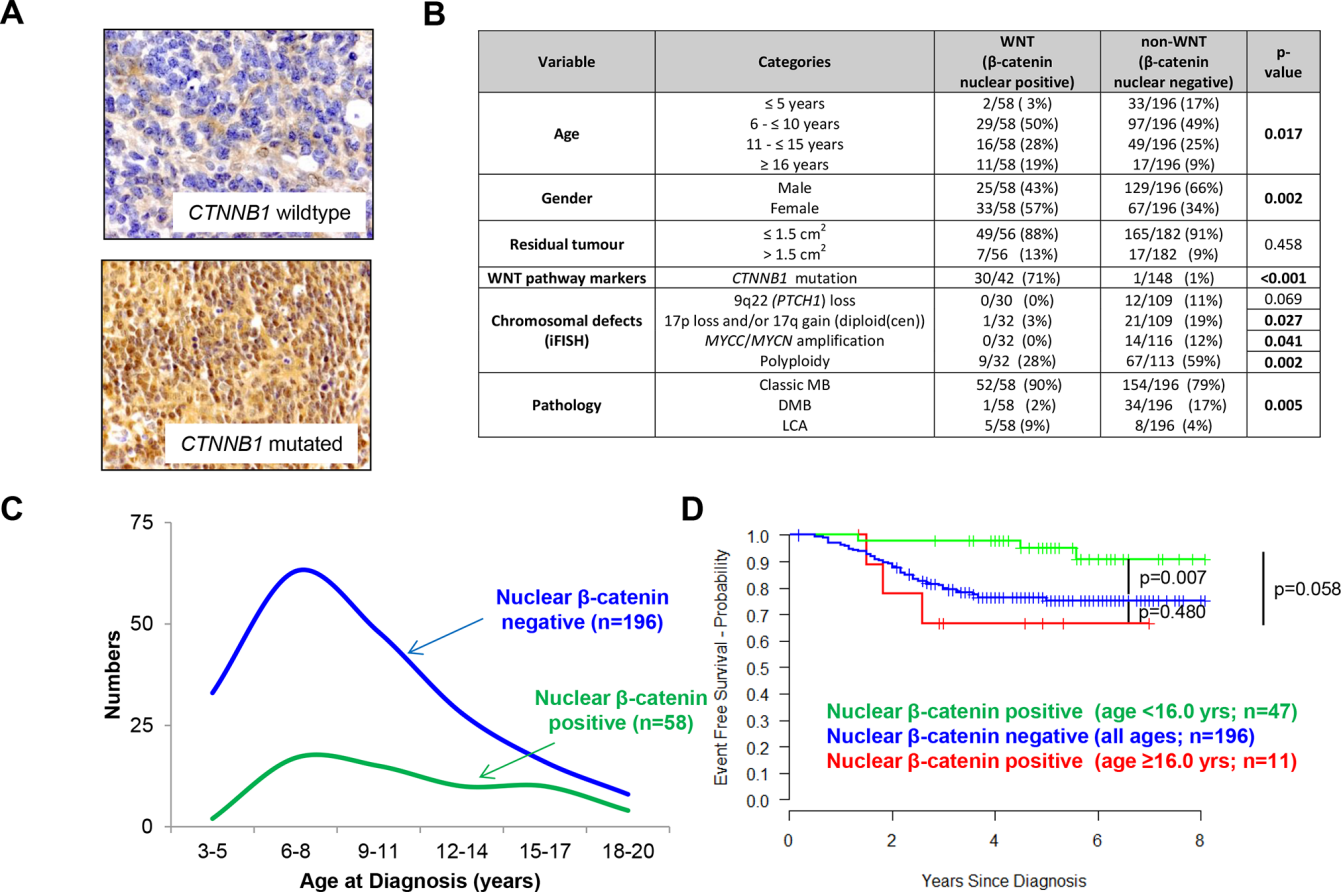
MB_WNT_ subgroup tumors: clinical, pathological and molecular correlates **A.** Examples of tumors scored negative and positive for β-catenin nuclear accumulation, with *CTNNB1* status also indicated. **B.** Distribution of clinical, histopathological and molecular markers between the MB_WNT_ (nuclear β-catenin accumulation) and non-MB_WNT_ medulloblastoma subgroups. **C.** Age distributions of MB_WNT_ and non-MB_WNT_ patients. **D.** Kaplan-Meier plots and associated ‘*p*’ values (log-rank test) shown for MB_WNT_ patients with age at diagnosis younger than 16 years, older than 16 years versus non-MB_WNT_ cases.

22.8% (58/254) of assessable tumors were MB_WNT_ positive by β-catenin IHC [10] (Figure 1A). 15.9% of tumors (31/195) harbored *CTNNB1* activating mutations (Supplementary Figure 1); all except one were observed in tumors with strong nuclear protein accumulation (*n = 30*; *p < 0.001*; Figure 1B). A further 12 tumors displayed β-catenin positivity (28.6% (12/42)) in the absence of *CTNNB1* exon 3 mutation.

MB_WNT_ subgroup tumors were clinically, pathologically and molecularly distinct [11]: All except one displayed CMB or LCA histology (*p = 0.005*), and the group displayed gender parity in contrast to the male predominance in non-MB_WNT_ patients (*p = 0.002*). All copy number aberrations (CNAs) tested were infrequent or absent in MB_WNT_ and polyploidy was less frequent (*p = 0.002*; Figure 1B). MB_WNT_ patients showed a broader age distribution than non-MB_WNT_, with 11/58 (19.0%) MB_WNT_ patients ≥16.0 years old at diagnosis, suggesting a secondary peak in adolescents/young adults (Figure 1B, 1C).

**Figure 2:**
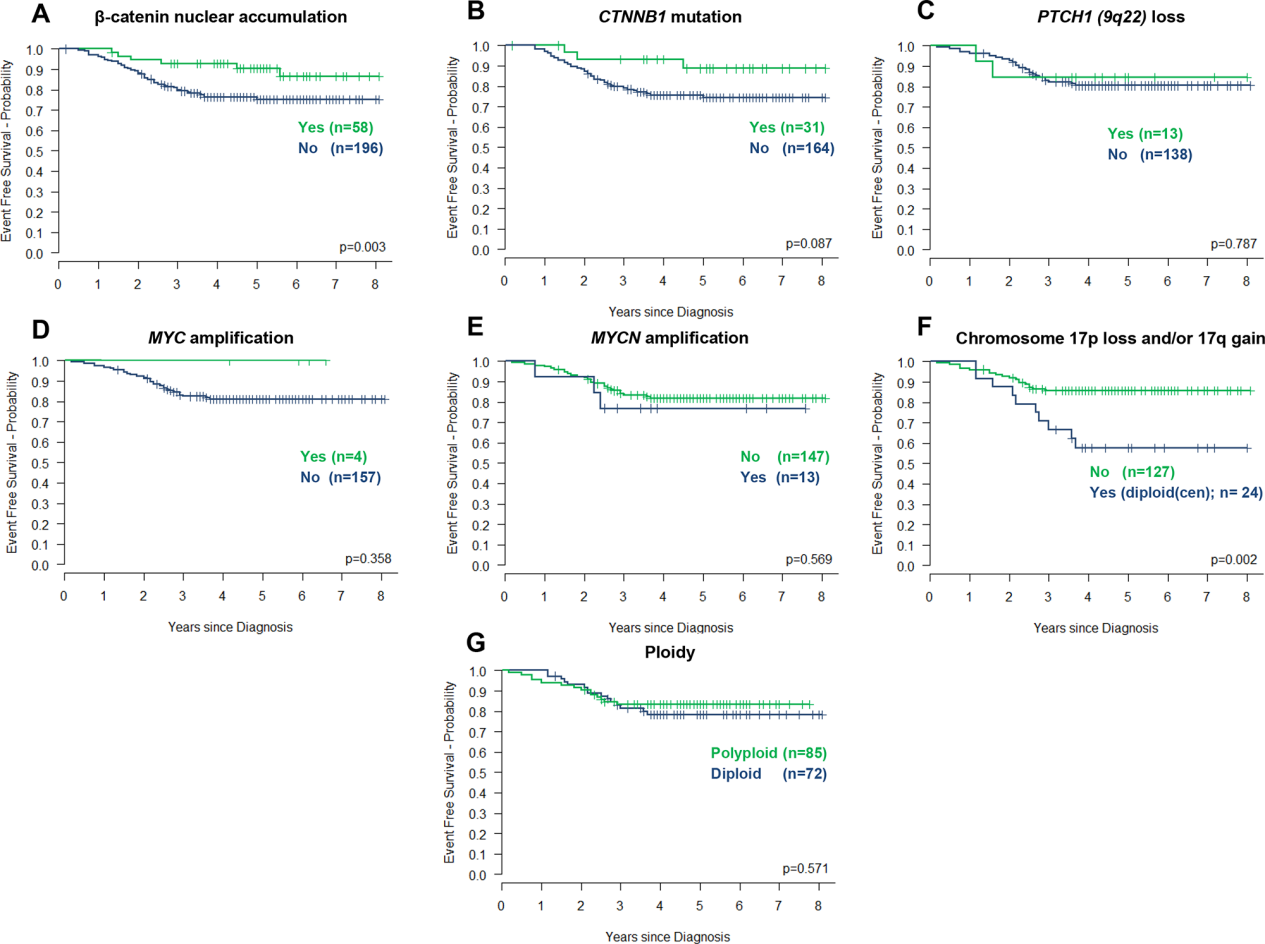
Prognostic significance of molecular disease features in the HIT-SIOP-PNET4 cohort Kaplan-Meier plots and associated ‘*p*’ values (log-rank test) are shown for each feature. Chromosome 17 losses and gains observed against a diploid centromeric reference score (diploid(cen)) are represented.

Favorable outcomes for patients with MB_WNT_ tumors were confirmed in univariate analysis, (Table 1, Figure 2 (*p = 0.019*, Cox proportional hazards test; *p = 0.003*, log-rank test)). *CTNNB1* mutation did not reach significance (Table 1, Figure 2A, 2B). 6/58 (10.3%) MB_WNT_ tumors relapsed; all had β-catenin nuclear accumulation in >50% of cells and 3/4 assessed tumors harbored a *CTNNB1* mutation (Supplementary Figure 2A). Of note, the EFS rate in MB_WNT_ patients aged ≥16.0 years at diagnosis appeared lower than in MB_WNT_ patients <16.0 years (*p = 0.058*; Figure 1D). Direct comparison of patients aged below 16.0 years from the HIT-SIOP-PNET4 and SIOP-UKCCSG-PNET3 trials showed equivalent five-year survival rates (±SE) for MB_WNT_ patients (0.951 (±0.034) vs. 0.877 (±0.058); *p* = 0.434), but significantly better outcomes (0.754 (±0.034) vs. 0.671 (±0.036); *p* = 0.033) in non-MB_WNT_ patients, and reduced frequencies of clinical high-risk features (M+ and/or R+ disease), in the HIT-SIOP-PNET4 cohort (Supplementary Figure 3).

### Copy number aberrations

*MYC* (2.5% (4/161) tumors tested) and *MYCN* (8.1% (13/160)) amplifications detected by iFISH were mutually exclusive and were detected in non-MB_WNT_ disease (Figure 1). Neither was associated with previously reported high-risk clinical (residual tumor) or pathological (LCA tumors included in this study) [1] disease features, or an adverse prognosis (Table 1, Figure 2D, 2E). iFISH and qPCR estimations of *MYC/MYCN* copy number were concordant in 90% of tumors analyzed by both methods (*n = 131*). Polyploidy (54% (85/157)) and *PTCH1* (9q22) losses (10.6% (13/151)) were not associated with prognosis (Table 1, Figure 2C, 2G).

**Figure 3:**
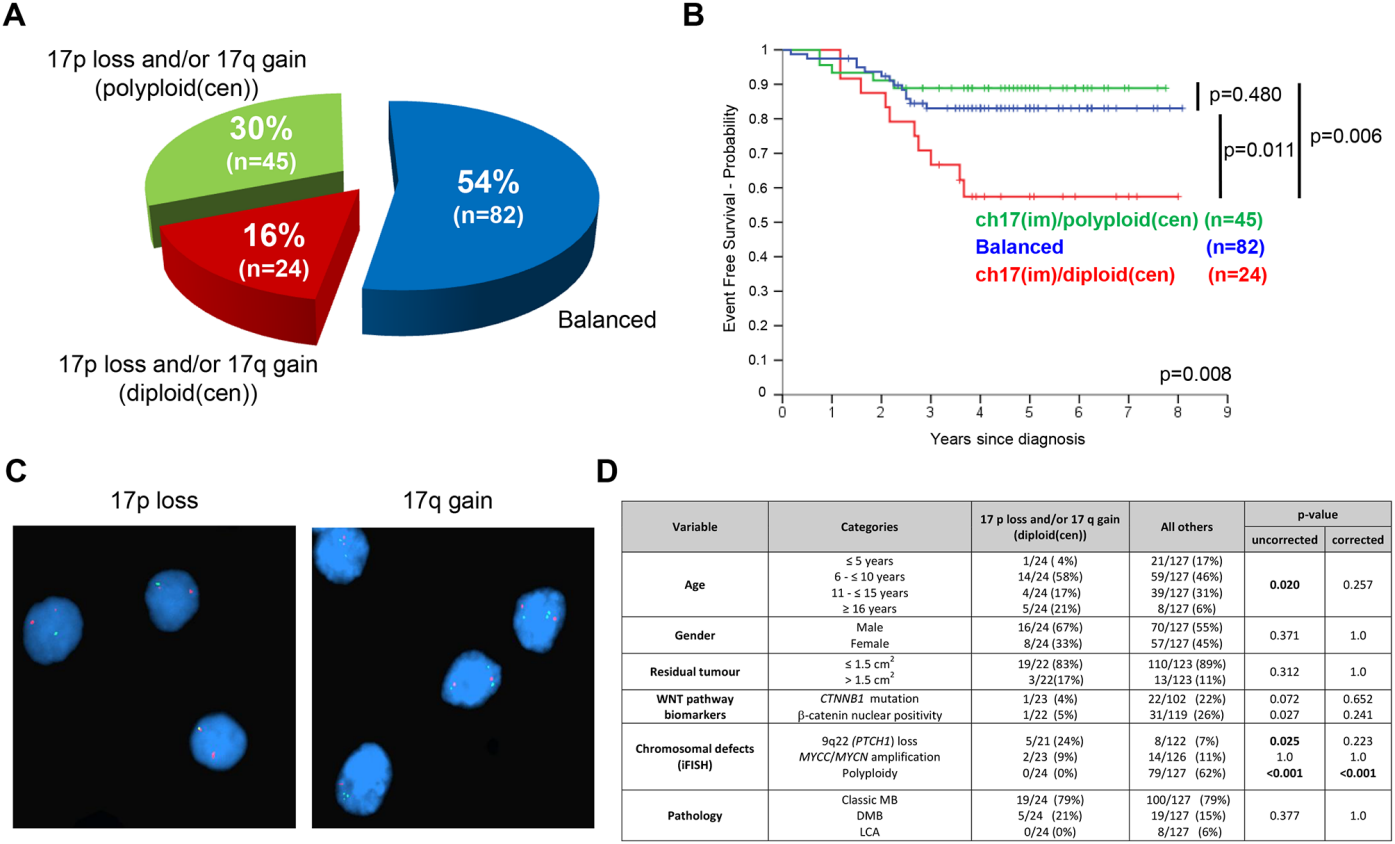
Chromosome 17 defects in HIT-SIOP-PNET4 cohort tumors Patterns **(A, C)** and prognostic significance (**B.**; ‘p’, log-rank tests) of chromosome 17 defects detected by iFISH. **C.** iFISH analysis showing (**i**) 17p loss (single green signals) and (**ii**) 17q gain (three green signals) against a diploid centromeric background (two red signals). Nuclei are counterstained blue. **D.** Relationship of ch17(im)/diploid(cen) defects to clinico-pathological and molecular disease features assessed (‘p’, Fisher's exact or χ^2^ tests; corrected and uncorrected values are shown). Abbreviations: ch, chromosome; im, imbalance (p-gain and/or q-loss); diploid(cen), diploid centromeric signal; polyploidy(cen), polyploidy centromeric signal.

Chromosome 17 imbalances (q-arm gains and/or p-arm losses) were frequent (45.7% (69/151) tumors assessed). Imbalances were molecularly heterogeneous (Figure 3), and occurred against diploid (2 signals) and polyploid (>2 signals) centromeric reference backgrounds (Figure 3A, 3C). Strikingly, this heterogeneity was clinically significant. Tumors with chromosome 17 imbalances/diploid background (ch17(im)/diploid(cen); 16% (24/151)) were significantly associated with a poor outcome (*p < 0.007*; Table 1, Figure 2F), while tumors with imbalances/polyploid background (ch17(im)/polyploid(cen); 30% (45/151)) were not, and behaved equivalently to balanced tumors (Figure 3B). Ch17(im)/diploid(cen) (*n = 24*) most commonly involved p-arm loss (to a single copy) in conjunction with q-arm gain (17/24), consistent with isochromosome (17q), but isolated p-arm losses (4/24) and q-arm gains (3/24) also contributed. This tumor group peaked in children 6–10 years at diagnosis and all but one tumor was found in non-MB_WNT_ disease, but the group was not associated with other clinico-pathological factors (Figure 3D). Notably, the prognostic significance of ch17(im)/diploid(cen) was gender-specific and 8/9 relapses in this group occurred in male patients (Supplementary Figure 4).

### Clinical and biological prognostic factors: Multivariate analysis

Multivariate analyses were performed separately on the clinically-defined non-metastatic (i.e. M0) and standard-risk (i.e. M0/R0) patient cohorts within the HIT-SIOP-PNET4 study; these analyses were based on all features showing significance in univariate analysis (*p* < 0.05; Table 1, Figure 2). To account for all features not having been assessed in all patients, we performed this multivariate analysis on different patient groups, based on the data available (Table 2). In the non-metastatic patient cohort, R+ disease was independently significant in all analyses, while MB_WNT_ and time to radiotherapy were either significant or marginally significant, consistent with findings from the univariate analysis. In the standard-risk cohort (i.e. following removal of R+ patients), ch17(im)/diploid(cen) was the only feature independently and significantly predictive of a poor outcome (Table 2).

**Table 2:** Multivariate analysis of independent risk factors in the HIT-SIOP-PNET4 cohort, shown for patients within the clinically-defined non-metastatic (M0, R+/R0) disease and standard-risk (M0, R0) patient groups

Non-metastatic (M0, R+/R0) disease										
Variable	Category	Cohort 1 (*n* = 315) Data available: Residual tumor status, Time to radiotherapy			Cohort 2 (*n* = 238) Data available: Residual tumor status, Time to radiotherapy, β-catenin status			Cohort 3 (*n* = 136) All data available		
		*n*	HR (± CI)	*p*	*n*	HR (± CI)	*p*	*n*	HR (± CI)	*p*
**Residual tumor**	≤1.5 cm^2^ >1.5 cm^2^	284 (90%) 31 (10%)	1.0 **2.74 (1.41–5.34)**	**0.003**	214 (90%) 24 (10%)	1.0 **3.08 (1.47–6.48)**	**0.003**	120 (88%) 16 (12%)	1.0 **3.14 (1.17–8.45)**	**0.023**
**Time to radiotherapy**	Continuous	315	**1.03 (1.01–1.06)**	**0.004**	238	**1.04 (1.01–1.07)**	**0.022**	136	1.04 (1.00–1.09)	0.076
**β-catenin nuclear accumulation**	No Yes	Data not available			182 (76%) 56 (24%)	1.0 **0.37 (0.16–0.88)**	**0.025**	104 (76%) 32 (24%)	1.0 0.25 (0.06–1.12)	0.070
**17p loss and/or 17q gain (diploid(cen))**	No Yes	Data not available			Data not available			115 (85%) 21 (15%)	1.0 1.95 (0.79–4.83)	0.149

Standard-risk (M0, R0) disease							
Variable	Category	Cohort 1 (*n* = 214) Data available: Time to radiotherapy, β-catenin status			Cohort 2 (*n* = 120) All data available		
		*n*	HR (± CI)	*p*	*n*	HR (± CI)	*p*
**Time to radiotherapy**	Continuous	214	1.01.03 (0.99–1.07)	0.111	120	1.03 (0.97–1.08)	0.339
**β-catenin nuclear accumulation**	No Yes	165 (77%) 49 (23%)	1.0 0.45 (0.17–1.15)	0.096	93 (78%) 27 (22%)	1.0 0.22 (0.03–1.69)	0.145
**17p loss and/or 17q gain (diploid(cen))**	No Yes	Data not available			103 (86%) 17 (14%)	1.0 **3.66 (1.40–9.52)**	**0.008**

Patients with central radiological and pathological review were analyzed (*n* = 315). Variables which displayed statistical significance in univariate analysis (Table 1) were tested in separate multivariate analyses based on patients with available data. Statistically significant independent risk-factors and associated hazard ratios are marked in bold type. HR, hazard ratio; CI, 95% confidence interval.

### Risk stratification models for standard-risk medulloblastoma

**Figure 4:**
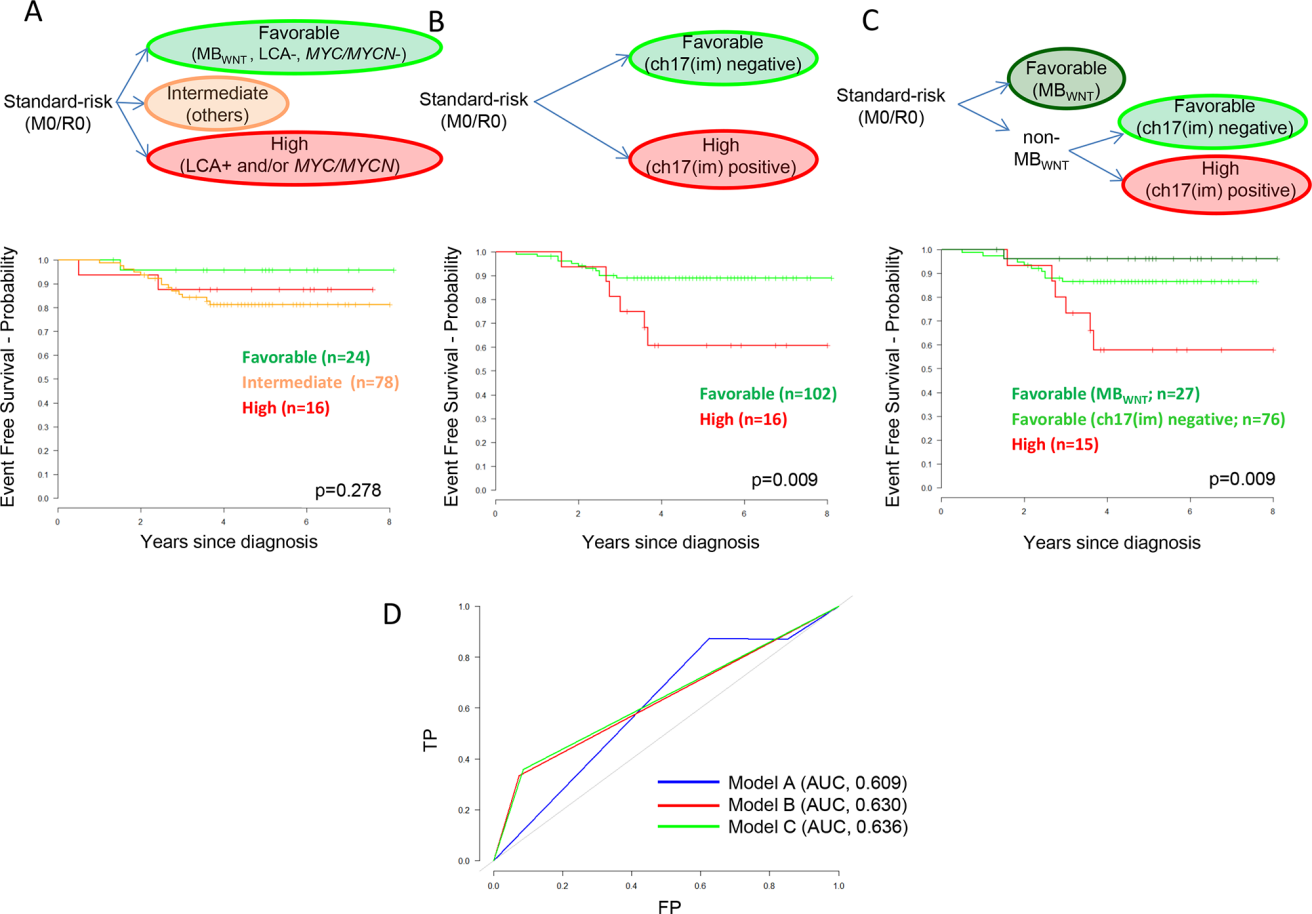
Biomarker-driven risk-stratification models for standard-risk (M0/R0) medulloblastoma based on patients from the HIT-SIOP-PNET4 cohort with data available for all parameters (*n = 118*) **A.** Established disease-wide survival model for non-infant medulloblastoma [1, 10, 14] (LCA pathology and/or *MYC/MYCN* amplified, high-risk; MB_WNT_ and no high-risk features, favorable-risk; others, intermediate-risk). **B.** Empirically-derived survival model for non-infant, standard-risk medulloblastoma. **C.** Illustrative survival model for non-infant, standard-risk medulloblastoma, incorporating the distinction of MB_WNT_ patients into the empirically-derived model. Kaplan-Meier plots and associated ‘*p*’ values (log-rank tests) show EFS. M0, non-metastatic; R0, no significant post-surgical tumor residuum; ch17(im), ch17(im)/diploid(cen) tumors. **D.** Time-dependent receiver operator characteristic (ROC) curves showing predictive performance of the three models for survival at five-years, determined as the area-under-curve (AUC). TP, true positive; FP, false positive. Chromosome 9 defects were not assessed in survival modelling due to missing data points.

The standard-risk patient group, defined by M0/R0 disease, forms the basis of current clinical trials [1]. We therefore next used our data to develop risk-stratification models for this patient group. First, we generated Cox models from all variables (listed in Table 1) using 90% of patients in our cohort, and selected the most significant model to predict survival for the remaining 10% of patients, using a 10-fold cohort re-selection strategy for cross-validation. Ch17(im)/diploid(cen) was selected as the sole prognostic feature in every fold, thus forming a model for the prediction of poor outcome within the standard-risk disease group (61 ± 13% vs. 89 ± 3% survival at five-years, *p = 0.009*) (Figure 4). In each fold, the model was not improved by the addition of any other covariate. We next compared model performance in our standard-risk cohort, against the current clinico-biological stratification scheme for the SIOP-PNET5 trial [1], which was defined previously based on disease-wide non-infant cohorts [1, 10, 14] (Figure 4A). Survival prediction at 5-years using the new cross-validated model improved performance and increased the area-under-curve (AUC) from 0.609 to 0.630 in ROC curve analysis (Figure 4D), and allowed 86% (102/118) of patients to be classified into a favorable-risk group, compared to 20% (24/118) in the established model. In view of the established favorable prognosis of MB_WNT_ tumors [1, 3, 10], confirmed in our cohort, we assessed the impact of MB_WNT_ status within this new model (Figure 4C). Inclusion was not detrimental to model performance (Figure 4D), however five-year survival for the favorable-risk MB_WNT_ (96 ± 4%) and non-MB_WNT_ ch17(im)/diploid(cen) negative (87 ± 4%) patient groups were not significantly different (*p* = 0.189). Finally, findings were equivalent when patients aged up to 16.0 years at diagnosis were considered in isolation (Supplementary Figure 5).

## DISCUSSION

The prospective assessment within HIT-SIOP-PNET4 of disease-relevant biomarkers, with reported significance in ≥2 previous retrospective series, alongside clinical and pathological factors, has provided important new insights to biomarker-driven risk stratification in clinically-defined standard-risk medulloblastomas. Post-operative residual tumor, delayed radiotherapy and MB_WNT_ were validated as independent prognostic factors. Importantly, in the standard-risk (M0/R0) disease group, which forms the basis of current clinical trials [1], the use of distinct biomarkers (chromosome 17 status determined at the cellular copy-number level) and novel survival models allows the improved stratification of disease-risk. Moreover, we have established first mechanisms for prospective pan-European biological studies as a basis for the assessment of biomarker-driven therapies, and future therapeutic advances.

Assessment of MB_WNT_ subgroup patients in the HIT-SIOP-PNET4 cohort revealed significant new insights to their clinical behavior, and comparison to other trials-based studies, to support future trial design. Their favorable prognosis was validated, supporting their consideration for individualized risk-stratified therapies; notably, despite equivalent EFS rates in the MB_WNT_ groups, a significantly higher EFS was observed for the non-MB_WNT_ group in our non-metastatic HIT-SIOP-PNET4 cohort (75% five-year EFS) compared to previous studies of the SIOP-UKCCSG-PNET3 trials cohort which included high-risk patients [2, 3]. MB_WNT_ relapses were observed at higher frequency in patients with delayed radiotherapy or aged >16.0 years at diagnosis (Figure 1D; Supplementary Figure 2). Patients >16.0 years were not ascertained in our previous analyses of SIOP-UKCCSG-PNET3 which defined the favorable prognosis of the MB_WNT_ group [2, 10], and the present data are consistent with the bi-modal age distribution and worse prognosis reported for adults compared to children within MB_WNT_ in retrospective series [4, 17, 18]. Although the incidence of such cases is too low to draw firm conclusions, these observations thus indicate patient age >16.0 years and delayed radiotherapy negatively influence survival in MB_WNT_, and indicate radiotherapy is important component of its multi-modal treatment. Similarly, only 1 event was observed in MB_WNT_ tumors also displaying previously reported high-risk disease features (MB_WNT_/R+ (*n = 7*), MB_WNT_/LCA (*n* = 5)) supporting their favorable prognosis following standard-risk therapy. *CTNNB1* mutation rates in MB_WNT_ tumors, defined by β-catenin IHC, were equivalent to SIOP-UKCCSG-PNET3 (30/42 (71.4%) and 20/31 (64.5%), respectively [10]) and our data did not support reduced survival rates in mutation-negative MB_WNT_ (Supplementary Figure 2A).

Study design, and the prospective collection of clinical material and biological data, was undertaken in 2000–2010. This limited our ability to assess in the PNET4 cohort the other non-MB_WNT_ molecular subgroups, on which consensus emerged in 2012 [11]. Insufficient FFPE-derived tumor material remained following our planned prospective analysis (see Materials and Methods; typically <200 ng double-stranded (ds)DNA [measured by picogreen fluorometric quantitation] and ≤1 4 μm section remaining), to enable subgroup assessment using established methods (approximately 1 μg FFPE-extracted dsDNA required for DNA methylation array analysis [14, 19, 20]; >2 sections for IHC-based assignment to MB_SHH_ subgroup [21]).

From our findings, high-risk biomarkers previously validated in disease-wide studies appear to have different prognostic relevance in the standard-risk clinical disease group, from which tumors with high-risk clinical features (e.g. M+ disease) have been excluded. In this clinical context, *MYC/MYCN* amplification and LCA were not associated with each other, with clinical high-risk factors (one *MYCN*/R+ and one *MYCN*/LCA tumor were observed) or with poor prognosis when observed in isolation as risk-factors in the clinically-defined non-metastatic/standard-risk group reported. These findings are consistent with observations based on SIOP-UKCCSG-PNET3 (which included high-risk patients), where *MYC/MYCN* amplification were associated with LCA pathology and were prognostic in high-risk (i.e. when observed in tumors from patients with other high-risk disease features) but not standard-risk disease [7, 10]. In most recent genomics studies, *MYC*-amplified tumors were most commonly observed in MB_Group3_, while *MYCN*-amplified tumors were associated with MB_SHH_ (where they were also associated with *TP53* mutation and LCA) and MB_Group4_, but were only prognostic in MB_SHH_ [12, 14, 22–24]. Our findings may thus reflect (i) the numbers of *MYC/MYCN* amplified tumors observed in this cohort, or (ii) biological heterogeneity and/or subgroup-dependency, including the trial amendment to cease recruitment of patients with LCA tumors (see methods), potentially limiting ascertainment of tumors with interactions between these factors. Evaluation/outcome monitoring of further patients defined by these features will now be required, prior to any refinements of their prognostic relevance used in the design of future clinical studies.

Our findings fully support the continued consideration of R+ patients as high-risk, and their exclusion from the standard-risk disease group [1]. Most notably, ch17(im)/diploid(cen) was the strongest independent biomarker risk-factor in M0/R0 standard-risk disease, characterizing a patient group with <60% five-year EFS; however, imbalances against a polyploid background were not significant. This previously undisclosed and clinically-significant heterogeneity observed between chromosome 17 imbalanced tumors is important, and these data suggest the biological impact of different patterns of chromosome 17 imbalance is equivalently heterogeneous. Variable prognostic associations have been reported for chromosome 17 imbalances in previous large studies [6, 10]; however, the complex patterns of imbalance revealed by iFISH analysis of individual tumor cells in this study were either not investigated or not detectable using the whole-biopsy copy number methodologies (e.g. array-CGH, SNP array) employed in many previous studies [9, 24–28]. Chromosome 17 imbalances are predominantly observed in MB_Group3_ and MB_Group4_ [24] and, in whole-biopsy SNP-array based investigations, Shih et al. [12] recently reported that the association between iso (17q) and a poor prognosis was restricted to MB_Group3_ patients. Concerted studies are now required to reconcile these findings and establish the relationship between tumor subgroup, cellular patterns of chromosome 17 imbalance, and prognosis.

Following designation of ch17 (im)/diploid (cen) tumors as high-risk in cross-validated survival models of standard-risk patients within the HIT-SIOP-PNET4 cohort, the EFS of remaining patients did not differ significantly from the MB_WNT_ group, allowing the classification of >80% of patients into a favorable-risk category. This model outperforms established prognostication schemes in our standard-risk cohort.

Alongside methods developed for testing chromosome 17 imbalances at the cellular level in routinely-collected tumor material, these findings provide a straightforward scheme for risk-stratification in the clinically homogeneous group of children with standard-risk medulloblastoma, and a strong basis for their validation and further investigation in future clinical trials of this group. Future study concepts must ensure collection of sufficient FFPE alongside high-quality biological material (e.g. snap-frozen, histologically-controlled tumor tissue), from large patient numbers, to support further biomarker discovery and validation, including understanding their behavior in the context of the consensus medulloblastoma expression / DNA methylation subgroups.

## MATERIALS AND METHODS

### Patient cohort, pathological review, material collection & processing

338 patients with non-metastatic (M0 [29]) medulloblastoma, treated in 120 European centers and 11 countries, were enrolled on HIT-SIOP-PNET4 over a 6 year period (2001–2006) [15]. Patients were randomized to receive post-operative treatment with either hyper-fractionated (HFRT) or conventionally fractionated/standard (STRT) radiotherapy and were followed up for a median of 4.8 years; all patients received the same chemotherapy. Clinical features of the cohort have been reported [15] (summarized in Table 1). The two treatment arms showed no significant difference in 5-year event-free survival (EFS) [15], and were considered together for biological analysis.

Post-operative radiological review was undertaken for 317/338 (93.7%) patients, the remainder were reviewed locally. Patients without significant post-surgical tumor residuum (≤1.5 cm^2^; R0) were defined as standard-risk [1]. A histopathological diagnosis of medulloblastoma was confirmed by five neuropathologists (including DF-B and TP), who performed central reference review of all patients. Tumors were classified using WHO criteria [30], and assigned to the classic (CMB), desmoplastic/nodular (DMB) or large-cell/anaplastic (LCA) sub-entities [16]. LCA were defined by a predominant component of tumor cells with either characteristic large-cell or severe anaplastic cytology, or both [8, 16, 31, 32]. DMB showed a significant tumor component with reticulin fiber-free islands (nodules) surrounded by reticulin fiber-rich tumor areas. Reactive fiber induction (‘desmoplastic reaction’, e.g. due to leptomeningeal growth) did not qualify as DMB [16, 33]. A study amendment was made in November 2003 to not enroll further LCA tumors, on the basis of their reported poor prognosis [8, 15, 31, 32].

During sample processing for reference pathology, excess tumor material was collected for biological studies where available. Two slides (4 μm tissue sections) were prepared for β-catenin immunohistochemistry (IHC). In addition, two tubes with 4 × 20 μm sections were collected, one to isolate nuclei for interphase fluorescence *in situ* hybridization (iFISH) analysis, the other for genomic DNA extraction (*CTNNB1* mutation, *MYC/MYCN* qPCR analysis (see below)). DNA was extracted using the QIAamp DNA Mini Tissue Kit (Qiagen GmbH, Düsseldorf, Germany) according to the manufacturer's instructions.

### Assessment of copy number aberrations

Tumor nuclei were isolated and CNAs of reported biological or prognostic significance assessed by iFISH using probes specific for *MYCN* (2p24) and *MYC* (8q24) (amplification previously associated with poor prognosis and LCA pathology [5–7, 10, 21]), *PTCH1* (9q22; loss associated with the sonic hedgehog medulloblastoma molecular subgroup (MB_SHH_) and DMB [5, 6, 11, 17, 21]), and the p- and q- arms of chromosome 17 (imbalances associated with a poor prognosis [5, 6]), alongside reference probes to the chromosome 2, 8, 9 and 17 centromeric regions, as previously described [5, 10]. Signals in >200 non-overlapping nuclei were scored to give region of interest: centromere signal ratios for individual cells. For chromosome gains and losses, the modal score was considered representative of genetic status. *MYC* or *MYCN* gene amplification was defined by double-minute patterns or homogeneously staining regions in ≥5% of nuclei [5, 7, 10]. Tumor ploidy was determined as the modal status of the four centromeric probes (>2 signals at ≥2 probes, polyploid) assessed. Quantitative PCR (qPCR) was used to estimate *MYC* and *MYCN* gene copy numbers, as described [34, 35].

### MB_WNT_ subgroup status

Assessment of β-catenin nuclear accumulation by IHC has been widely studied as an MB_WNT_ biomarker [9, 14, 36–38], and was performed as described using the monoclonal anti-β-catenin antibody 14 (Transduction laboratories) [2, 10, 39]. Tumors with >10% positive nuclei were scored positive (nuclear accumulation); the same cut-off as used in the published SIOP-UKCCSG-PNET3 cohort [10]. The few tumors showing nuclear accumulation in single cells (typically <5%) were classified negative. For *CTNNB1* mutation analysis, exon 3 was PCR-amplified from tumor DNA using the primers 5′-GATTTGATGGAGTTGGACATGG-3′/5′-TGTTCTTGAGTGAAGGACTGAG-3′, and sequenced using standard methods [39].

### Prospective biological studies: molecular biomarker assessment

Overall, biological data were collected prospectively from FFPE tumor material for 269/338 (79.5%) patients. Data collection rates varied according to the individual assays and the amount of tissue available. Notably, the success rate of sampling for β-catenin IHC did not differ between centers recruiting low (≤2; 77% success) and high (>7; 80%) patient numbers. Cases with available biological data from each assay were distributed randomly across the major disease demographics were thus considered representative of the whole cohort for further analysis (Supplementary Table 1).

### Statistical analysis

EFS was defined as time from diagnosis to recurrence, progression or death during remission (of any reason). Patients not experiencing an event were censored at last follow-up. The database and biological data collection for this analysis was closed July 1^st^ 2010. Kaplan–Meier curves, log-rank tests and unadjusted Cox proportional hazards models were used to test univariate EFS markers. Adjusted Cox proportional hazards models were used to test for independent disease-risk markers. For univariate and multivariate survival analyses which included ‘time to radiotherapy’ (as a continuous variable), EFS times were landmarked to the commencement of radiotherapy. Risk stratification models for standard-risk medulloblastoma were developed in the cohort of M0/R0 patients with data available for all prognostic parameters (*n = 118*). This cohort was demographically representative of the entire standard-risk cohort within HIT-SIOP-PNET4 (*n* = 286; Supplementary Table 2). Associations between clinico-pathologic and/or molecular variables were examined using Fisher's exact or X^2^ tests, as appropriate. *P*-values were corrected for multiplicity using the Bonferroni method where indicated. Analysis was performed using SPSS (SPSS, Chicago, U.S.A.) and R [40] software. Data proportions presented in Tables and Figures are based on patients with available data and may not add to 100% due to rounding.

## SUPPLEMENTARY FIGURES AND TABLES


