# Supplementary material for: Biomarker-driven stratification of disease-risk in non-metastatic medulloblastoma: Results from the multi-center HIT-SIOP-PNET4 clinical trial
Source: Oncotarget. 2015 Sep 5;6(36):38827–39. doi: 10.18632/oncotarget.5149 (PMC4770740; doi:10.18632/oncotarget.5149)
Supplement: Supplementary file 1 [file oncotarget-06-38827-s001.pdf]

SUPPLEMENTARY FIGURES AND TABLES

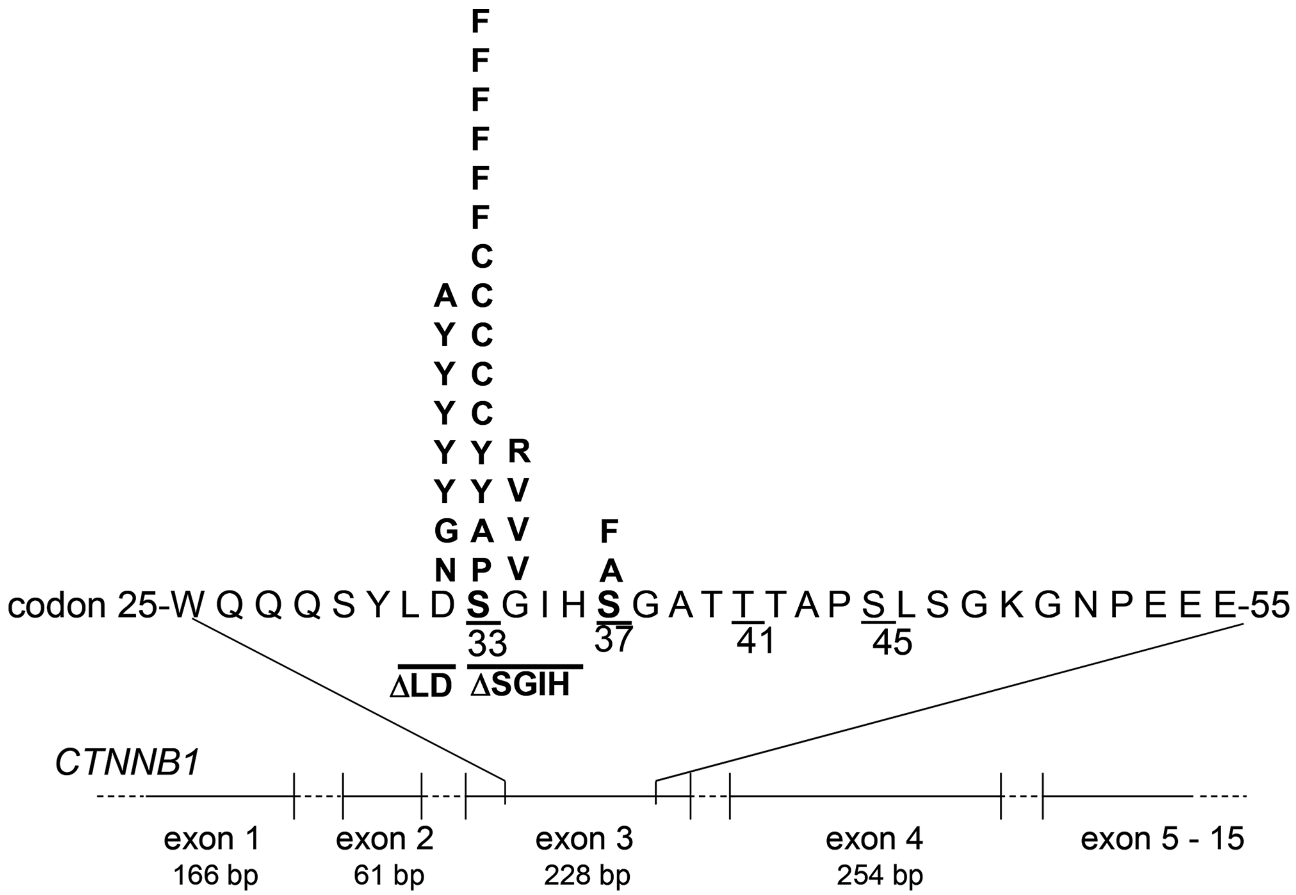

Supplementary Figure S1: Schematic diagram of the distribution of *CTNNB1* exon 3 mutations. 29 point mutations and two in-frame deletions clustering around the serine phosphorylation sites 33 and 37 were detected by Sanger sequencing.

A

Patient	β-catenin accumulation	*CTNNB1* mutation	Gender	Age (years)	Randomisation arm	Time to radiotherapy (days)	Residual tumor	Pathology variant	Chromosome 17 status	*MYC* amplified	*MYCN* amplified	Time to relapse (years)
1	Yes	No	F	16.0	STRT	**57**	**>1.5cm²**	Classic	Balanced	0	0	2.6
2	Yes	Yes	F	9.8	STRT	**54**	≤1.5cm²	Classic	NA	NA	NA	4.5
3	Yes	Yes	M	16.1	STRT	**58**	≤1.5cm²	Classic	Balanced	0	0	1.5
4	Yes	Yes	M	16.3	HFRT	32	≤1.5cm²	Classic	NA	NA	NA	1.8
5	Yes	NA	M	8.7	STRT	36	≤1.5cm²	Classic	NA	NA	NA	1.3
6	Yes	NA	F	13.8	HFRT	43	≤1.5cm²	Classic	NA	NA	NA	5.6

B

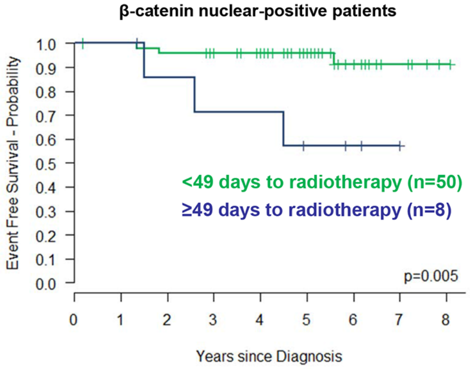

Supplementary Figure S2: A. Clinical, pathological and molecular characteristics of relapsing patients with β-catenin nuclear accumulation. High-risk factors identified in this cohort are marked in bold. M, Male; F, Female; NA, data not available. **B.** Prognostic significance of time to radiotherapy for patients with MB_{WNT} tumors. A Kaplan-Meier plot and associated '*p*' value (log-rank test) is shown.

A

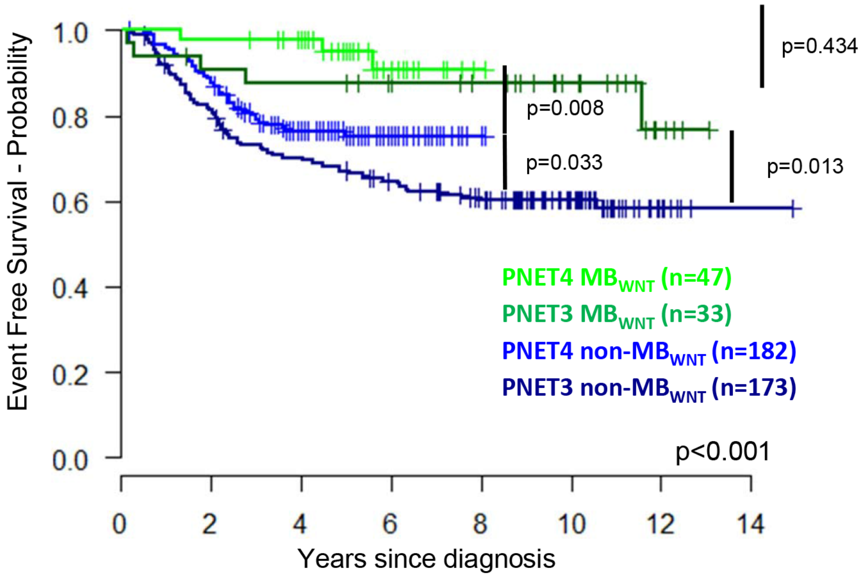

B

Variable	Categories	PNET4 Cohort (n=226)	PNET3 Cohort (n=206)	*p*
MB_{WNT}	Yes<br>No	47<br>179	33<br>173	0.22
Metastatic disease	M-<br>M+	226<br>0	169<br>37	**2.0×10^{-13}**
LCA pathology	Yes<br>No	12<br>214	18<br>188	0.19
Residual tumor	≤1.5 cm^2<br>>1.5 cm^2<br>No data	194<br>18<br>14	105<br>98<br>3	**$<2.2 \times 10^{-16}$**

Supplementary Figure S3: Comparison of EFS in MB_{WNT} and non-MB_{WNT} patients from the HIT-SIOP-PNET4 and SIOP-UKCCSG-PNET3 trials. A. Kaplan-Meier plots and associated '*p*' values (log-rank test) are shown for each group. **B.** Incidence of high-risk clinical and pathological features in each cohort. *P* values from Fisher Exact tests are shown. Data for SIOP-UKCCSG-PNET3 comprised patients aged below 16.0 years at diagnosis and are taken from Ellison et al (2011) [10]; comparisons were made using the age-matched group from HIT-SIOP-PNET4. MB_{WNT} status was determined using β-catenin IHC, with identical methods and criteria, in both trials. Missing data were excluded before making comparisons.

A

Patient	Chromosome 17 status (all diploid (cen))	Gender	Age (years)	Randomisa-tion arm	Time to radiotherapy (days)	Residual tumor	Patholgy variant	β-catenin nuclear accumulation	*c-MYC* amplified	*MYCN* amplified	Time to relapse (years)
**1**	17p loss and 17q gain	M	6.4	STRT	31	≤1.5 cm²	Classic	No	No	No	3.6
**2**	17p loss and 17q gain	M	6.7	HFRT	23	≤1.5 cm²	Classic	No	No	No	1.6
**3**	17p loss and 17q gain	M	7.2	STRT	38	≤1.5 cm²	Classic	No	NA	No	1.2
**4**	17p loss	M	7.5	HFRT	20	**>1.5 cm²**	DMB	NA	No	No	1.2
**5**	17q gain	M	7.8	STRT	27	≤1.5 cm²	DMB	No	No	No	2.8
**6**	17p loss and 17q gain	F	8.8	STRT	23	≤1.5 cm²	Classic	No	No	No	3.0
**7**	17p loss and 17q gain	M	9.4	STRT	39	≤1.5 cm²	DMB	No	No	No	2.7
**8**	17p loss and 17q gain	M	9.8	HFRT	32	**>1.5 cm²**	Classic	No	No	No	2.2
**9**	17p loss and 17q gain	M	20.6	STRT	41	≤1.5 cm²	Classic	No	No	No	3.7

B

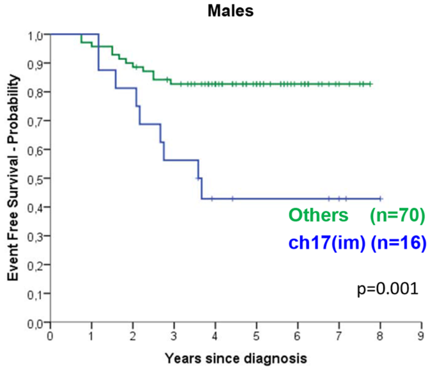

C

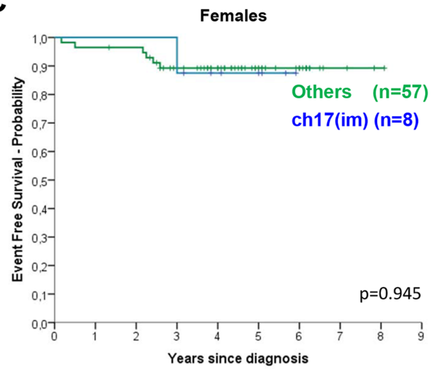

Supplementary Figure S4: A. Clinical, pathological and molecular characteristics of relapsing patients with ch17(im)/diploid(cen) tumors. High-risk factors identified in this cohort are marked in **bold**. M, Male; F, Female; NA, data not available. **B, C.** Prognostic significance of ch17(im)/diploid(cen) tumors in male (B) and female (C) patients. Kaplan-Meier plots and associated '*p*' values (log-rank test) are shown. Abbreviations: ch, chromosome; im, imbalance p-loss and/or q-gain diploid(cen), diploid centromeric signal.

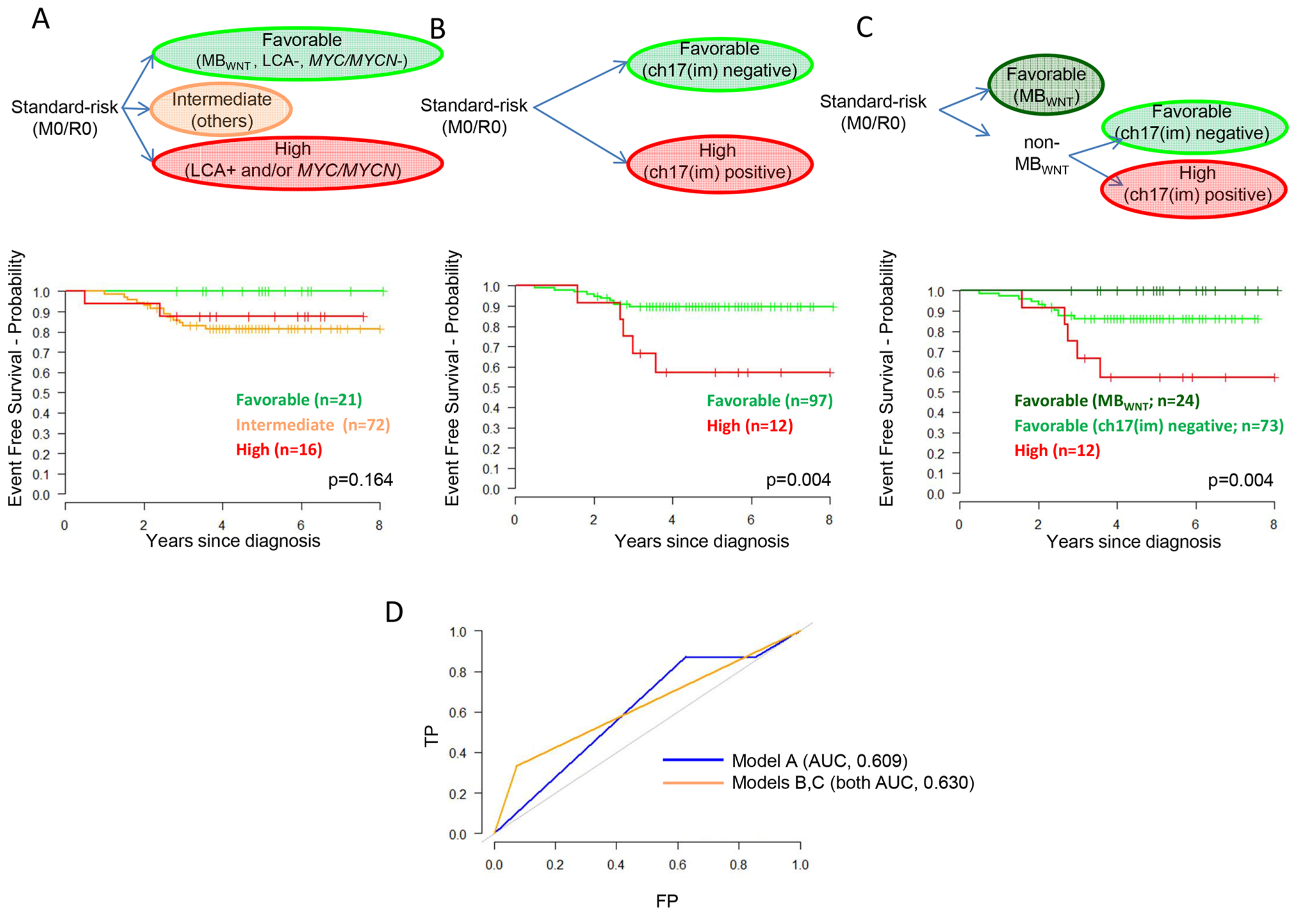

Supplementary Figure S5: Biomarker-driven risk-stratification models for standard-risk (M0/R0) childhood medulloblastoma based on patients aged 3.0 to 16.0 years at diagnosis from the HIT-SIOP-PNET4 cohort, with data available for all parameters (*n* = *109*). A. Established disease-wide survival model for non-infant medulloblastoma [1, 10, 14] (LCA pathology and/or *MYC/MYCN* amplified, high-risk; MB_{WNT} and no high-risk features, favorable-risk; others, intermediate-risk). **B.** Empirically-derived survival model for non-infant, standard-risk medulloblastoma. **C.** Illustrative survival model for non-infant, standard-risk medulloblastoma, incorporating the distinction of MB_{WNT} patients into the empirically-derived model. Kaplan-Meier plots and associated '*p*' values (log-rank tests) show EFS for. M0, non-metastatic; R0, no significant post-surgical tumor residuum; ch17(im), ch17(im)/diploid(cen) tumors. **D.** Time-dependent receiver operator characteristic (ROC) curves showing predictive performance of the three models for survival at five-years, determined as the area-under-curve (AUC). TP, true positive; FP, false positive. Chromosome 9 defects were not assessed in survival modelling due to missing data points.

Supplementary Table S1: Demographics of the HIT-SIOP-PNET4 patient cohort and biomarkers assessed.

Variable	Categories	Cohort	β-catenin IHC	*CTNNB1* mutation	Genomic markers (qPCR)	Genomic markers (iFISH)				
					MYC and *MYCN*	*MYC*	*MYCN*	Ch17	*PTCH1* (9q22)	Ploidy
**Material collected for analysis (% of cohort)**		338	254 (75%)	195* (58%)	187* (55%)	205 (61%)				
**Material successfully assessed (% of cohort)**			254 (75%)	195 (58%)	183 (54%)	161 (47%)	160 (47%)	151 (45%)	151 (45%)	157 (46%)
**Gender**	Male	211(62%)	154	121	116	95	95	86	88	92
	Female	127 (38%)	100	74	67	66	65	65	63	65
**Age at diagnosis**	≤5 years	46 (14%)	35	22	22	25	25	22	23	24
	6 to ≤10 years	175 (52%)	126	96	93	80	79	73	74	77
	11 to ≤15years	83 (24%)	65	55	47	43	43	43	42	43
	≥16 years	34 (10%)	28	22	21	13	13	13	12	13
	Mean/Median	9.6/9.0	9.8/9.0	10.0/9.0	9.9/9.0	9.5/9.0	9.5/9.0	9.6/9.0	9.5/9.0	9.5/9.0
	Min-Max	4–20	4–20	4–20	4–20	4–20	4–20	4–20	4–20	4–20
**Pathology**	Classic	273 (80%)	206	151	137	126	125	119	118	123
	DMB	47 (14%)	35	32	37	26	26	24	25	26
	LCA	16 (5%)	13	12	9	9	9	8	8	8
	No review	2 (1%)	–	–	–	–	–	–	–	–
**Residual tumor**	≤1.5 cm^2	286 (85%)	214	167	159	138	137	129	130	135
	>1.5 cm^2	31 (9%)	24	22	18	17	17	17	16	17
	No data	21 (6%)	16	6	6	6	6	5	5	5

*Samples with sufficient DNA extracted to initiate analysis. Ch, chromosome.

Supplementary Table S2: Demographics of the standard-risk (M0/R0) cohort used for risk modelling do not differ significantly from the complete standard-risk cohort within HIT-SIOP-PNET4.

Variable	Categories	Complete standard-risk cohort (*n* = 286)	Standard-risk cohort: Survival modelling (*n* = 118)	*p*
**Gender**	Male	179 (63%)	66 (56%)	0.22
	Female	107 (37%)	52 (44%)	
**Age at diagnosis**	≤5 years	39 (14%)	17 (14%)	0.81
	6 to ≤10 years	155 (54%)	60 (51%)	
	11 to ≤15years	66 (23%)	32 (27%)	
	≥16 years	26 (9%)	9 (8%)	
	Mean/Median	9.4/9.0	9.5/9.0	
	Min-Max	4–20	4–20	
**Pathology**	Classic	232 (81%)	94 (79%)	0.82
	DMB	39 (14%)	19 (16%)	
	LCA	13 (5%)	5 (4%)	
	No review	2 (1%)	–	

The representativeness of the survival modelling cohort was tested against the complete cohort using Fisher's Exact test and X^2 test as appropriate. Incidence of demographic features, and their percentage are given.
